# Supplementary material for: Patterns of Intron Gain and Loss in Fungi
Source: PLoS Biol. 2004 Nov 30;2(12):e422. doi: 10.1371/journal.pbio.0020422 (PMC532390; doi:10.1371/journal.pbio.0020422)
Supplement: Table S1 — Also available at http://genes.mit.edu/NielsenEtAl/. (4.3 MB ZIP). [file pbio.0020422.st001.zip › NielsenEtAl/html/40.html]

AN5008.1.NCU02293.1.MG06573.1.FG10793.1


```
 CLUSTAL W (1.82) Multiple Sequence Alignments - Introns Inserted


Sequence 1: NCU02293.1	117 aa
Sequence 2: MG06573.1	114 aa
Sequence 3: FG10793.1	134 aa
Sequence 4: AN5008.1	115 aa
Alignment Length: 138 aa
Number Identitical Residues: 27 aa
Alignment Score (without introns) 1935


MG06573.1 	MALLGLFK-SEDEKRADELRQGTAVPKRAERQRCWDSRDKYFACLDKAGIIDALKDDKAA
NCU02293.1	-MVLGLFEQSEADKRAAEVRAGTVAPTRSERQKCWMARDGYFACLDRNNIIDALKDEKAA
FG10793.1 	---MGLWP-FSSESRADAIRSGDAIPNRQERKICWAARDAYFNCLDANNIVDATKDPSAT
AN5008.1  	---MGWFPWSSSDS-NKASDGGRIAPDRTSRQRCWIGRDQFFACLDDNDIVDSIKGDKEA
          	   :* :   . :.       *   * * .*: ** .** :* ***  .*:*: *. . :

MG06573.1 	AKACKSESNEFEQNCAAQW0VTHFKKYRLANYQKEQRLKALEAQGAVKMDSQVNFK----
NCU02293.1	AKACKAESAEFEKDCAAQW0VTYFKKWRVQDIQKKARLKELEAQGAVRMDVNTDFKARE-
FG10793.1 	KRACPTETVDFERDCAAAW~VKYFKQWRVADIQKKRRLDELRAQGAQEMTVTTSFAPEGG
AN5008.1  	RKKCAKEIAEFEAACSSTW0VKYFKEKRVMEYNRDKTIERIKKEDAATVRDLKSQGWSPK
          	 : *  *  :**  *:: * *.:**: *: : ::.  :. :. :.*  :    .      

MG06573.1 	-------------------
NCU02293.1	-------------------
FG10793.1 	AGEKGKGVDDIQAMLNKKK
AN5008.1  	-------------------
          	
```
